# Microclimate Impacts Survival and Prevalence of *Phytophthora ramorum* in *Umbellularia californica*, a Key Reservoir Host of Sudden Oak Death in Northern California Forests

**DOI:** 10.1371/journal.pone.0098195

**Published:** 2014-08-06

**Authors:** Matthew V. DiLeo, Richard M. Bostock, David M. Rizzo

**Affiliations:** Department of Plant Pathology, University of California Davis, Davis, California, United States of America; Virginia Tech, United States of America

## Abstract

*Phytophthora ramorum*, an invasive pathogen and the causal agent of Sudden Oak Death, has become established in mixed-evergreen and redwood forests in coastal northern California. While oak and tanoak mortality is the most visible indication of *P. ramorum*’s presence, epidemics are largely driven by the presence of bay laurel (*Umbellularia californica*), a reservoir host that supports both prolific sporulation in the winter wet season and survival during the summer dry season. In order to better understand how over-summer survival of the pathogen contributes to variability in the severity of annual epidemics, we monitored the viability of *P. ramorum* leaf infections over three years along with coincident microclimate. The proportion of symptomatic bay laurel leaves that contained viable infections decreased during the first summer dry season and remained low for the following two years, likely due to the absence of conducive wet season weather during the study period. Over-summer survival of *P. ramorum* was positively correlated with high percent canopy cover, less negative bay leaf water potential and few days exceeding 30°C but was not significantly different between mixed-evergreen and redwood forest ecosystems. Decreased summer survival of *P. ramorum* in exposed locations and during unusually hot summers likely contributes to the observed spatiotemporal heterogeneity of *P. ramorum* epidemics.

## Introduction

Sudden Oak Death, caused by the oomycete *Phytophthora ramorum*, has killed potentially millions of native oak (*Quercus* spp.) and tanoak (*Notholithocarpus densiflorus*) trees in Oregon and northern California since the mid-1990s [1], [2]. This invasive pathogen has become established primarily in two forest types: mixed-evergreen forests, dominated by coast live oak (*Quercus agrifolia*), and redwood forests, dominated by redwood (*Sequoia sempervirens*) and tanoak. These two forests types occur in a fine-scale mosaic that is superimposed on the diverse topography of this region. *P. ramorum*-infested forest sites are most commonly found within 30 km of the Pacific Coast and San Francisco Bay, where mild winter wet seasons and plentiful fog provide a conducive environment for sporulation and infection of aerial host tissues [1]–[3].

The most dramatic symptoms of *P. ramorum* infection include bole cankers on oaks and both bole cankers and shoot dieback on tanoaks, leading to mortality and stem failure of mature trees [4]. Relatively few spores are produced from infected tanoaks, however, and most California oak species appear to provide an epidemiological dead end for this pathogen [2], [5]. Instead, the establishment and reproduction of *P. ramorum* in California forests is primarily driven by California bay laurel (*Umbellularia californica*), an evergreen reservoir host that is common in both mixed-evergreen and redwood forests [5], [6]. *P. ramorum* causes a minor leaf blight on bay laurel, often infecting a large proportion of the leaves in the lower, visible canopy, yet does not appear to cause significant injury to the host [7]. Infected bay laurel leaves are capable of producing many thousands of splash-dispersed sporangia and zoospores during the winter wet season and appear to be the primary vehicle by which the pathogen survives the summer dry season [5], [8], [9].

The viability of *P. ramorum* in infected bay laurel leaves has been found to decrease progressively during the summer dry season in California forests [3], [10]. One previous study [3] also documented a more pronounced decrease in pathogen viability over the summer in bay laurel leaves from a mixed-evergreen forest relative to a nearby redwood forest. While these two sites did not differ in temperature or humidity, the water potential of infected trees was found to be lower in the mixed-evergreen forest [3]. In the present study, the survival of *P. ramorum* within infected bay laurel leaves was measured over three summers coincident with the collection of ecophysiological and microclimate data in order to identify environmental conditions that inhibit over-summer survival of the pathogen. An improved understanding of the over-summer survival will help define the ecological limits of *P. ramorum* and inform Sudden Oak Death risk management.

## Materials and Methods

### Research site locations and characteristics

Sites were located in the Sonoma and Mayacmas mountain ranges within Sonoma County, CA. This region is subject to a Mediterranean climate, consisting of cool, wet winters and hot, dry and intermittently foggy summers. Vegetation occurs as a patchwork of annual grasslands, mixed-evergreen forests and redwood forests. 12 sites were selected from a larger set of existing long-term study sites outfitted with weather-monitoring data loggers [11]. These 12 sites were chosen to represent the diverse topographic, climatic and ecological environments in which bay trees are found while allowing sufficient access to trails and roads as required for water potential measurements. Three to nine bay laurel trees with ramorum blight symptoms were tagged at each site and followed through the duration of the study. A total of 25 bay laurel trees were tagged in each of mixed-evergreen and redwood forest. An initial set of 18 trees was tagged in the spring of 2005 and the remaining 32 trees were tagged in the spring of 2006. Mixed-evergreen forests were located within Fairfield Osborn Preserve (FOP, 122° 35′41″W, 38° 20′37″N), Sugarloaf Ridge State Park (SRSP, 122° 31′41″W, 38° 26′30″N) and Annadel State Park (ASP, 122° 62′36″W, 38° 42′92″N). Redwood forests were located in Jack London State Historic Park (JLSHP, 122° 33′16″W, 38° 21′2″N) and the Sonoma Development Center (SDC, 122° 32′10″W, 38° 20′52″N). All field work was completed under permits issued by California State Parks (SRSP, ASP, JLSHP, SDC), Sonoma State University (FOP), and California Department of Food and Agriculture. Field measurements were made in July, August, September and October during 2005, 2006 and 2007.

### Microclimate and physiological measurements

Data loggers were used to record the temperature and humidity of the air at each site hourly [Onset Corporation, Bourne, MA, USA; Campbell Scientific, Logan, UT, USA] [R. Meentemeyer, UNC-Charlotte, unpublished]. All sensors were placed approximately 1 m above the ground and were shielded from the sun by the forest canopy or a protective solar shield to prevent inflated temperature measurements. Most tagged trees were located within a few meters of their corresponding data logger and all were within 200 m. Canopy coverage above each tree was manually estimated by eye. Aspect and slope were measured at the base of each tree and were used to calculate insolation, an estimation of the amount of solar radiation that an area of land receives based on the angle of the land relative to the position of the sun [12]. Elevation data were obtained from topographic maps [R. Meentemeyer, UNC-Charlotte, personal communication]. Vapor pressure deficit was calculated from temperature and relative humidity data. Average vapor pressure deficit, average temperature and the number of days where temperature exceeded 30°C were all calculated for each 30 day period preceding each time point.

Leaf water potential of individual bay laurel leaves was measured with a pump-up pressure chamber [PMS Instrument Company, Albany, OR, USA]. All measurements were obtained between 11 AM and 1 PM during a three-day window surrounding each time point in order to minimize the influence of short term weather variation. This restriction limited the number and location of potential study sites; while representative of local environments, all sites were relatively easily accessed by vehicle and foot. Leaf water potential measurement procedures were adapted from McCutchan and Shackel [13]. Variation in leaf water potential within a given canopy at a given time point was generally 0.01 MPa or less. Minimum leaf water potential, occurring in August or September, was recorded for each tree each year.

### Pathogen isolation and quantitative image analysis

*P. ramorum* commonly causes tip and edge necrosis on bay laurel leaves that consumes 5–10% leaf area. Ten symptomatic leaves were arbitrarily selected for removal from each tree at each time point. Symptomatic leaf images were captured with a scanner and analyzed with APS Assess© [American Phytopathological Society, St. Paul, MN]. The total leaf area and percent necrotic area was recorded. Subsequently, half of each necrotic lesion (including adjacent chlorotic and symptomless leaf tissue) was cut from each leaf and plated on the selective medium PARP (cornmeal agar with pimaricin, ampicillin, rifampicin, and pentachloronitrobenzene) [14]. *In vitro* hydration treatments did not increase isolation frequency, as may occur when chlamydospores are present [9]. Excised lesion margins were sampled from all parts of each symptomatic leaf, including necrotic spots in the interior of the leaf when present. Leaves were not surface sterilized. Following a 3–4 week incubation at 18°C, *P. ramorum* mycelia were identified by colony morphology and the presence of large chlamydospores [1]. It is assumed that the inability to isolate *P. ramorum* from symptomatic leaves is solely due to the death of the pathogen, therefore pathogen survival was recorded where *P. ramorum* was successfully isolated from symptomatic leaf material. This is supported by previous work, which found isolation frequency to be well correlated with pathogen viability, as measured with RT-PCR [15]. Although other *Phytophthora* species are known from bay laurel in California forests [24], no other *Phytophthora* species were isolated from bay laurel leaves in this study.

### Data analysis

Statistical analysis was performed in three stages. First, systemic differences between mixed-evergreen and redwood forest sites were tested. Leaf water potential, percent canopy cover, elevation and insolation were compared between bay laurel trees located in mixed-evergreen and redwood forest sites using the Wilcoxon rank sum test [JMP IN 5.1 (Cary, NC)]. Variation in leaf area, lesion area, percent lesion area, average temperature, average vapor pressure deficit and number of days exceeding 30°C were compared between trees located in mixed-evergreen and redwood forest sites and over the summer using repeated measures ANOVA.

Associations between these ecophysiological variables and *P. ramorum* survival were assessed using a linear mixed effects model [16]. *P. ramorum* survival, the percentage of symptomatic leaves from which *P. ramorum* was successfully isolated at each time point, was arcsine square root transformed. Year was included as a blocking factor, while month, number of days exceeding 30°C, leaf water potential, percent canopy coverage and lesion area were included with main effects. Redundant measured variables were omitted to avoid biasing the final model. Thus, elevation was omitted as it is primarily associated with differences in temperature. Temperature was included only as the number of days that exceeded 30°C, as this has been shown to be directly detrimental to *P. ramorum* [17]. Vapor pressure deficit was omitted as it is strongly associated with leaf water potential, a more direct measurement of leaf water status. Percent canopy coverage was included instead of insolation as the former was judged to be a better proxy for the amount of full sun that each canopy was exposed to. Finally, lesion area was included while percent leaf area was omitted as the ability of the pathogen to survive the summer may be influenced by the mass of mycelium or number of propagules within the leaf. Random effects were included for both site and tree nested within site. Correlation was allowed among the measurements for each tree within each year to account for possible dependence, assuming compound symmetry. Interaction effects were not examined due to the complexity of the model. Model assessment was performed using graphical analysis of residuals and a Shapiro-Wilks test for normality. Statistical significance was declared at the 0.05 level. All analyses were implemented using the MIXED procedure in SASR 9.1 for Windows (Cary, NC).

Finally, each variable found to have a significant correlation with *P. ramorum* survival was further analyzed by linear regression. This analysis was done on averaged data for each site. The average percent *P. ramorum* survival calculated for each site in October was compared to the average minimum leaf water potential, the average percent canopy coverage and the average number of days exceeding 30°C at each site. *r^2^* values were calculated for each regression using Microsoft Office Excel 2003 (Redmond, WA).

All data sets used in this study are available from http://datadryad.org/resource/.

## Results

### *P. ramorum* survival

**Figure 1:**
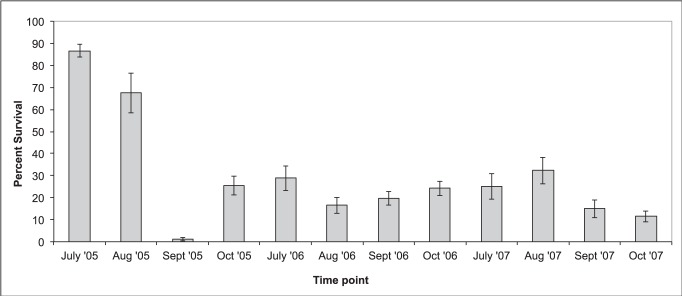
Average percent survival of *P. ramorum* in symptomatic bay laurel leaves across three years. The percent of symptomatic bay laurel leaves from which the pathogen could be recovered declined during the summer of 2005, as has been documented in previous studies, but remained low for the following two years. Specific weather events occurring in the study region may account for the observed pattern (± standard error).

The percentage of symptomatic bay laurel leaves that contained viable *P. ramorum* infections fell from 87% in July 2005 to 25% in September 2005 and remained below 33% for all time points in 2006 and 2007 (Figure 1). Over three summers, *P. ramorum* survived within an average of 24% of symptomatic bay laurel leaves from mixed-evergreen forest sites and 26% of leaves from redwood forest sites. Average end of summer (October) *P. ramorum* survival was 16% in mixed-evergreen forest sites and 21% in redwood forest sites, a difference that is not statistically significant.

### Associations with forest type

Descriptive statistics were calculated for all trees and for trees partitioned by forest type (Table 1). Although the survival of *P. ramorum* did not differ significantly between mixed-evergreen and redwood forest sites, other variables did. Redwood forest sites had significantly less negative bay laurel leaf water potential (*P*<.0001), greater canopy cover (*P* = 0.0027) and lower elevation (*P*<.0001). Mixed-evergreen and redwood forest sites did not differ significantly by average temperature (*P* = 0.1262) or the number of days exceeding 30°C (*P* = 0.3925), although vapor pressure deficit was greater in mixed-evergreen forest sites (*P* = 0.0355). While bay laurel trees in redwood-forest sites had larger leaves (*P* = 0.0008) and larger lesions (*P* = 0.0004), the percent of the leaf consumed by necrosis did not vary between forest types (*P* = 0.4415). Lesion area and percent lesion area both decreased during the summer (P = 0.0002 and P<0.0001, respectively), while leaf area did not (*P* = 0.8612).

**Table 1:** Comparison of environmental variables between mixed-evergreen and redwood forest sites across three years.

Site Variables	Mixed-evergreen sites			Redwood sites			*p* value
	Range	Mean	S.E.	Range	Mean	S.E.	
Daily mean temperature (°C)	12.2–27.8	17.8	0.18	12.4–21.4	16.9	0.13	0.1262
Daily mean VPD (kpa)	0.4–2.1	0.8	0.02	0.3–1.2	0.7	0.01	0.0355
Number of days >30°C	0–17	4.3	0.27	0–13	3.3	0.22	0.3925
Elevation (m)	185–610	472	25	206–328	251	7	<.0001
Insolation (kWh/(m^2^d))	0.5–1.2	1.1	0.03	0.5–1.2	1.0	0.04	0.1307
Percent canopy cover	20–75	37	3	25–60	50	2	0.0027
Leaf water potential (MPa)	−1.4– −2.6	−2.1	0.1	−0.8– −2.2	−1.4	0.1	<.0001
Leaf area (cm^2^)	7.2–30.8	14.2	0.38	11.6–32.9	20.2	0.32	0.0008
Lesion area (cm^2^)	0.2–1.9	0.8	0.03	0.2–2.9	1.3	0.04	0.0004
Percent lesion area	1.0–20.5	6.0	0.31	1.8–15.3	6.6	0.21	0.4415

*p* values were generated from Wilcoxon rank sum tests and repeated measures ANOVA, as described in the Materials and Methods. S.E. indicates standard error.

### Mixed model ANOVA

Five variables were found to have significant associations with *P. ramorum* survival and were retained in the final model: year (*P* = .0074), month (*P* = .0153), leaf water potential (*P*<.0001), number of days exceeding 30°C (*P*<.0001) and percent canopy coverage (*P*<.0001) (Table 2). Lesion area (*P* = .9741) was not found to be significant. *P. ramorum* survival was positively associated with canopy cover and leaf water potential and negatively associated with the number of days over 30°C.

**Table 2:** Mixed model analysis of variance.

Effect	*F*	*P>F*
Year	5.16	0.0074
Month	3.53	0.0153
Leaf water potential	314.90	<.0001
Canopy cover	69.64	<.0001
Number of days above 30°C	15.76	<.0001
Lesion area	0.00	0.09741

Ecophysiological and site variables were associated with over-summer survival of *P. ramorum* in symptomatic bay laurel leaves.

### Post-hoc Analysis

Linear regression of *P. ramorum* survival by leaf water potential (*r^2^* = 0.21) and canopy cover (*r^2^* = 0.08) both showed positive correlations. Linear regression of survival by the number of days exceeding 30°C showed a negative correlation (*r^2^* = 0.35). Overall, survival of *P. ramorum* in symptomatic bay laurel leaves was associated with less negative water potential, greater canopy cover and fewer days with maximum hourly temperature over 30°C during the study period.

## Discussion

The percentage of symptomatic bay laurel leaves that contained viable *P. ramorum* infections decreased markedly during the summer of 2005 as previous work has suggested; falling from almost 90% to below 30% [3]. Although it was expected that this pattern would have been repeated in 2006 and 2007, survival instead remained below 33% for the remainder of this study. Such year-to-year variation in disease prevalence has been documented in previous studies of *P. ramorum* in these ecosystems. For example, prolonged spring rainfall, experienced in the study region in 2005, was linked to prolific sporulation from, and new infections of, bay laurel leaves well into June [3], [5]. In contrast, the much drier spring of 2004 had dramatically lower levels of sporulation and infection of bay laurel leaves [3], [5]. Likewise, the relatively dry spring in 2007 may partially explain the poor survival of *P. ramorum* observed in this year. In this context, the poor survival observed during the relatively wet spring of 2006 is surprising, though corroborated by an unusually early ceasing of *P. ramorum* sporulation in April (unpublished data). Whatever factors beyond rainfall limited survival and sporulation in the spring of 2006 was likely compounded by the intense heat wave that this region experienced during June of the same year. While in both 2005 and 2007, most sites had not been exposed to temperatures exceeding 30°C at all, in 2006 each site had experienced between 3 and 11 such days before the first survival time point was taken, possibly masking an earlier peak in infection viability in 2006.

The survival of *P. ramorum* within bay laurel leaves was negatively correlated with the number of days exceeding 30°C and positively correlated with percent canopy cover and leaf water potential. As these three variables inevitably interact, the statistical model cannot definitively distinguish between variables that are responsible for the death of the pathogen and those merely associated with it. However, the known biology of this system strongly implicates heat, exacerbated by solar heating, in the progressive death of *P. ramorum* within bay laurel leaves. It is very unlikely that water potential directly impacts the survival of *P. ramorum* in bay leaves as this and other *Phytophthora* species have been found to not only survive, but grow well at water potentials well below those recorded in this study [18], [19]. Alternatively, *in vitro* studies have found that exposure of *P. ramorum* to temperatures over 30°C decreases survival quantitatively over several hours [17]. Temperatures over 40°C, which occasionally were recorded in this study, have been shown to kill *P. ramorum* cultures in minutes to hours [17], [20]. Another study found that *P. ramorum* could be recovered specifically from chlamydospores and infected leaf disks over a week at 20 or 30°C, but that recovery quickly declined at 35°C and did not occur at 40°C [21]. Furthermore, it is important to consider that temperatures recorded in this study were obtained from sensors that were shielded from the sun. Sun exposure can quickly raise leaf temperatures up to 20°C above ambient in still air when little transpiration occurs, which is likely common during Mediterranean summers [22], [23]. It is interesting to note that for a few trees with canopies that were evenly split between high sun and deep shade environments, *P. ramorum* was consistently recovered at much higher proportions from leaves that were immersed in shade. Trees that were fully exposed to sun rarely produced successful isolations, and consequently, a few of these trees were not included in this final analysis due to a lack of symptomatic leaves at later time points (unpublished data).

Previous studies have confirmed that *P. ramorum* performs best in mild, wet and shaded environments, much like other foliar Phytophthoras [14]. The current study illustrates the critical role that microclimate plays in the survival of individual *P. ramorum* leaf infections through the summer dry season. While rainfall was not recorded during this study due to its absence in the summer dry season, it is clear that wet season rainfall also plays an important role in determining the prevalence of the pathogen during the summer months. Increased wet season rainfall has been associated with both increased inoculum production and pathogen survival in these forests and appears at least partially responsible for the greater inoculum production and infection observed in redwood-tanoak compared to mixed-evergreen forest sites [3]. Inoculum production and infection in mixed-evergreen sites appear particularly sensitive to wet season rainfall, as extended spring rains have been associated with levels of pathogen activity similar to that commonly seen in redwood-tanoak sites [5], [6]. Moreover, these extended spring rains may be required for *P. ramorum* epidemics to produce exponential growth, as commonly occurs in redwood-tanoak sites [3].

Just as a particularly conducive wet season is capable of supporting large amounts of sporulation and new infections, this study demonstrates that exposure to hot, sunny dry-season conditions appears capable of significantly reducing local *P. ramorum* populations, though not necessarily removing the potential for future epidemics [24], [25]. Such reductions in local *P. ramorum* populations likely contributes to delays in wet season epidemics and may ultimately limit pathogen prevalence at the beginning of the following dry season [3]. The implications of these seasonal dynamics on the long term spread and establishment of *P. ramorum* in changing California forest ecosystems remain to be seen.
